# The evolutionary history of the common bean (*Phaseolus vulgaris*) revealed by chloroplast and nuclear genomes analysis

**DOI:** 10.1007/s00122-025-04832-z

**Published:** 2025-02-08

**Authors:** Giulia Frascarelli, Teresa R. Galise, Nunzio D’Agostino, Donata Cafasso, Salvatore Cozzolino, Gaia Cortinovis, Francesca Sparvoli, Elisa Bellucci, Valerio Di Vittori, Laura Nanni, Alice Pieri, Marzia Rossato, Leonardo Vincenzi, Andrea Benazzo, Massimo Delledonne, Elena Bitocchi, Roberto Papa

**Affiliations:** 1 Department of Agricultural, Food and Environmental Sciences, Marche Polytechnic University, Via Brecce Bianche, 60131 Ancona, Italy; 2 Department of Biology, University Federico II of Naples, Complesso Universitario Monte Sant’Angelo, Naples, Italy; 3 Department of Agricultural Sciences, University of Naples Federico II, Via Università 100, 80055 Portici, Naples, Italy; 4 CNR-Institute of Agricultural Biology and Biotechnology, Via Edoardo Bassini 15, 20133 Milan, Italy; 5 Department of Biotechnology, University of Verona, Strada Le Grazie 15, 37134 Verona, Italy; 6 Department of Life Sciences and Biotechnology, University of Ferrara, 44100 Ferrara, Italy

## Abstract

**Key message:**

The origin of common bean was investigated throughout chloroplast and nuclear WGS data considering recombination events. Our results support the Mesoamerican origin of common bean.

**Abstract:**

The remarkable evolutionary history of the common bean (*Phaseolus vulgaris L.*) has led to the emergence of three wild main gene pools corresponding to three different eco-geographical areas: Mesoamerica, the Andes and northern Peru/Ecuador. Recent works proposed novel scenarios, and the northern Peru/Ecuador population has been described as a new species called *P. debouckii,* rekindling the debate about the origin of *P. vulgaris*. Here we shed light on the origin of *P. vulgaris* by analyzing the chloroplast and nuclear genomes of a large varietal collection representing the entire geographical distribution of wild forms including a large collection of Mesoamerican and Andean individuals. We assembled 37 chloroplast genomes de novo and used them to construct a time frame for the divergence of the genotypes under investigation, revealing that the separation of the Mesoamerican and northern Peru/Ecuador gene pools occurred ~ 0.15 Mya. Our results clearly support a Mesoamerican origin of the common bean and reject the recent *P. deboukii* hypothesis. These results also imply two independent migratory events from Mesoamerica to the North and South Andes, probably facilitated by birds. Our work represents a paradigmatic example of the importance of taking into account the genetic rearrangements produced by recombination when investigating phylogeny and of the analysis of wild forms when studying the evolutionary history of a crop species.

**Supplementary Information:**

The online version contains supplementary material available at 10.1007/s00122-025-04832-z.

## Introduction

Wild forms of the common bean (*Phaseolus vulgaris L.*) grow over a large geographical area in Latin America, from northern Mexico to northwestern Argentina (Toro Chica et al. 1990). The species has an intriguing evolutionary history that has resulted in at least three eco-geographical gene pools: Mesoamerican, Andean and northern Peru/Ecuador. The Mesoamerican gene pool is distributed in Mexico, Central America, Colombia and Venezuela, while the Andean genotypes spread from southern Peru to Bolivia and Argentina. Conversely, the northern Peru/Ecuador gene pool identified by Debouck (1986) is restricted to the western Andes. The first two populations include both wild and domesticated forms, whereas the northern Peru/Ecuador gene pool only has wild forms. Phylogeographical analysis, which emphasizes the close connection between genealogy and geography (Avise et al. 1987), has shown that the domestication of the common bean was preceded by two geographically distinct and isolated lineages (Mesoamerican and Andean). The genetic variability characteristics of wild relatives represent the primary resource for phylogenetics investigation. Indeed, domesticated genotypes underwent a genetic bottleneck due to the domestication process that resulted in a reduction of genetic variation and dramatic phenotypic changes (Meyer et al. 2012). Moreover, the extensive genetic diversity that characterize wild materials makes them an important plant genetic resource (PGR), and their study not only facilitates the sustainable conservation of the biodiversity but also is the foundation for the genetic improvement of crops (Salgotra and Chauhan 2023) since PGRs represent a source for desirable traits. Specifically, common bean is the main grain legume for human utilization, and the maintenance and the investigation of its genetic resources form the core development of both sustainable agriculture and a healthy food system (Bellucci et al. 2021).

Although phylogenetics is necessary to infer evolutionary relationships (Cavalli-Sforza and Edwards’ 1966), the rearrangement of genetic material during recombination events can produce artifacts (Schierup and Hein 2000). Indeed, recombination implies that different parts of a sequence can have distinct phylogenetic histories and can be related by more than one tree (Nordborg and Tavaré 2002). Furthermore, when considering recent divergence between species or populations, incomplete lineage sorting can cause further difficulties in the phylogenetic reconstruction and discordance between gene trees but also between nuclear and plastid trees. To address this issue, mitochondrial and chloroplast genomes have been extensively used to reconstruct the genetic lineages of animals and plants, respectively. Both organelles are haploid, implying a smaller population size than the nuclear genome, so organelle and nuclear genome data may trace different evolutionary histories. In plant genealogy, the chloroplast genome is widely preferred because it evolves more slowly than animal mitochondrial DNA but faster than plant mitochondrial DNA (Avise 2009).

The origin of the common bean is still debated, and numerous theories have been advanced. The northern Peru/Ecuador hypothesis suggested by Kami et al. (1995) was based on the type I seed storage protein phaseolin, which is characteristic of a central area in northern Peru/Ecuador (PhI). The lack of tandem repeats in this type of phaseolin implied its ancestral state and supported northern Peru/Ecuador as the center of origin. Evidence for a Mesoamerican origin was first provided by Rossi et al. (2009), who analyzed the genetic diversity of the Mesoamerican and Andean gene pools using amplified fragment length polymorphism markers. Bitocchi et al. (2012) provided further support by investigating the origin of the three wild gene pools using nuclear single-nucleotide polymorphisms (SNPs) at five independent loci, and their work was confirmed by the analysis of chloroplast simple sequence repeats (Desiderio et al. 2013). More recently, a speciation event occurring before the divergence of the Mesoamerican and Andean gene pools was hypothesized (Rendón-Anaya et al. 2017b). The speciation gave rise to the northern Peru/Ecuador population that has been described by the authors as a new species of *Phaseolus* named *Phaseolus deboukii* (Rendón-Anaya et al. 2017a). Based on the proposed Mesoamerican origin, Ariani et al. (2018) suggested the existence of a common ancestral population for the three gene pools that became extinct when the Mesoamerican and Andean gene pools diverged (“Protovulgaris hypothesis”). Those different hypotheses underline the presence of a research gap in understanding the origin of common bean. Specifically, a speciation event, proposed by the *P. debouckii* theory, would have implied a reproductive isolation of the northern Peru/Ecuador gene pool; nevertheless, the three common bean genetic groups belong to the primary gene pool (Singh 2001).

We therefore set out to elucidate the evolutionary history *of P. vulgaris* by investigating the relationships between the three major wild gene pools: Mesoamerican, Andean and northern Peru/Ecuador. We used chloroplast and nuclear sequence data of wild accessions, belonging to the three gene pools, to reconstruct the phylogeny of this species and to infer times of divergence among the three wild gene pools.

## Materials and methods

### Plant material, preparation of chloroplast/nuclear DNA libraries, and phaseolin extraction

Patterns of nucleotide variability were assessed across 97 *Phaseolus spp.* chloroplast DNA samples. These comprised 70 wild accessions of *P. vulgaris*, 22 of *P. coccineus*, three of *P. lunatus* and one of *P. acutifolius*, as well as one domesticated accession of *P. acutifolius*. The 70 *P. vulgaris* accessions covered the geographical distribution from northern Mexico to northwestern Argentina and included all three gene pools (44 Mesoamerican, 22 Andean and 4 northern Peru/Ecuador). The lower sampling number of the northern Peru/Ecuador accessions is due to the limited availability in the genebank of common bean genotypes carrying the type I of the Phaseolin protein (Debouck 1986). Ten *P. vulgaris* accessions were selected from the original panel for resequencing and nuclear genome analysis. The selection was based on geographical criteria, guaranteeing the representation of Mesoamerica, Andes and northern Peru/Ecuador and on the haplogroups identified by analyzing the chloroplast genome. Genomic DNA was extracted from the young leaves of single greenhouse-grown plants using the DNeasy Plant Mini Kit (Qiagen). Paired-end DNA libraries were constructed and sequenced from both ends using Illumina technology, with low coverage for the chloroplast genome samples and 10 × coverage for the nuclear genome samples. Seeds were provided by the United States Department of Agriculture (USDA) Western Regional Plant Introduction Station and the International Center of Tropical Agriculture (CIAT) in Colombia. To verify the presence of the ancestral type I phaseolin protein, phaseolin was extracted from seed samples of representative accessions and enriched as described in the Supplementary Methods.

### Reference mapping and SNP calling in the chloroplast dataset

Quality control was applied to raw reads before and after trimming using FastQC (Andrews et al. 2010). Trimmomatic v0.38 (Bolger et al. 2014) was used to remove Illumina technical sequences and filter out low-quality reads. Reads ≥ 75 nucleotides in length with a minimum Q-value of 20 were retained. FastQscreen was used for contamination screening (Wingett and Andrews 2018) and chloroplast reads were retrieved by screening the *P. vulgaris* nuclear (G19833) and chloroplast (NC_009259) genomes using Bowtie2 with default settings (Langmead and Salzberg 2012). The filtered reads were mapped to the chloroplast genome (NC_009259) using Bowtie2 with “local” settings, and the mapped reads were sorted and realigned with SAMtools v1.7 (Li et al. 2009). The read depth across the *P. vulgaris* chloroplast genome sequence was determined by using the BEDtools “genomecov” utility to find all uniquely mapping reads in the library (Quinlan and Hall 2010).

SNPs in the *P. vulgaris* chloroplast genome sequence were called using the “mpileup” utility of BCFtools (Li et al. 2009). All VCF files were merged using BCFtools and uninformative SNPs (singletons) were filtered out with VCFTOOLS (Danecek et al. 2011). The final set of SNPs was annotated with predicted functional effects using SnpEff (Cingolani et al. 2012). The VCF files were converted to Nexus and BAPS format using PGDSpider v2.1.1.5 (Lischer and Excoffier 2012).

### Reference mapping and SNP calling in the nuclear dataset

SNPs in the *P. vulgaris* nuclear genome sequence were called using the sequence_handling pipeline (Hoffman et al. 2018) available at the Minnesota Supercomputing Institute, followed by quality control using FastQC. Adapters were removed using Scythe v.1.2.8 (https://github.com/vsbuffalo/scythe) with default parameters (e.g., prior contamination rate of 0.05). Sequences were aligned to the *P. vulgaris* reference genome (accession G19833, v.2.1) using BWA-MEM v0.7.17 with default parameters (Li 2013; Poplin et al. 2017). The resulting SAM files were sorted and de duplicated, and read groups were added with Picard v2.4.1 (http://broadinstitute.github.io/picard/). Haplotypes were called using GATK v4.1.2 (Poplin et al. 2017) with a nucleotide sequence diversity estimate (Watterson theta value) of θW = 0.001. The latter was estimated using ANGSD (Sand Korneliussen et al. 2014) based on the samples and an ancestral sequence obtained by mapping *P. lunatus* reads to the *P. vulgaris* reference genome. The resulting gVCF files were used to jointly call SNPs on all samples. Hard-filtering was applied to increase the quality of the call-set (Danecek et al. 2021). Indels, non-biallelic sites, low-quality sites (missingness ≥ 50%) and sites with minor allele frequencies ≤ 0.01 were filtered. Finally, singletons were removed from the final set of SNPs to avoid noisy signals due to long-branch attraction effects. SNPs were annotated with SnpEff. Using the centromeric regions reported in Schmutz et al. (2014), a smaller set of SNPs was derived by selecting only those variants present in the pericentromeric regions which were defined by extending the centromeric regions 20% upstream and downstream.

### Genetic structure of the chloroplast dataset

The chloroplast SNP dataset was clustered using BAPS v6.0 (Cheng et al. 2013). We chose a mixture model due to the high probability that all markers were linked. Pairwise identity-by-state distances were estimated among all 97 samples and among the *P. vulgaris* accessions using PLINK v1.90b52 (Purcell et al. 2007) and the results were graphically represented by MDS. Haplotype network analysis was carried out using PopART (Leigh and Bryant 2015) with the TCS network (Clement et al. 2002).

### Assembly of chloroplast genomes

Full-length assembly was not possible for all 97 chloroplast genomes due to the low sequencing depth (Bedtools, “genomecov” utility). Therefore, we selected 31 *P. vulgaris* accessions based on coverage and geographical origin, representing all three gene pools: 19 Mesoamerican, 8 Andean and 4 PhI. We also included four samples of *P. coccieneus*, one of *P. acutifolius* and one of *P. lunatus*. NOVOPlasty v3.2 (Dierckxsens et al. 2017) was used for de novo genome assembly, seeded with the *P. vulgaris* sequences matk, accd, psbh, rrn16 and rpl32 (GenBank accession no. NC_009259).

### Phylogenetic analysis

An ML tree based on the 37 chloroplast genome de novo assemblies was computed using RAxML v8.1.2 (Stamatakis 2014) with the GTR substitution model, a bootstrap value of 10,000 and *P. lunatus* selected as the outgroup (Delgado-Salinas et al. 1999). The same analysis was applied to the concatenated SNPs, again with 10,000 bootstrap replicates. Trees were visualized using FigTree v1.4.4. An ML tree was also computed in RAxML for the nuclear dataset, only including those SNPs located in the pericentromeric regions. The SNPs were concatenated, and an individual ML tree was constructed for each of the 11 chromosomes, with 10,000 bootstrap replicates.

### Dating the divergence events

Molecular clock analysis was applied to the 37 chloroplast genome de novo assemblies. These were aligned using MAFFT (Katoh et al. 2018) to three *Vigna spp.* chloroplast genomes available in NCBI, namely *V. radiata* (NC_013843), *V. unguiculata* (NC_018051) and *V. angularis* (NC_021091), followed by Bayesian analysis in BEAST v2.6.2 (Bouckaert et al. 2014). The *BEAST method was used to produce the XML file, and the coalescent simulation was initiated by applying a relaxed log-normal molecular clock with a general time reversible model. The tree was calibrated using the divergence reported between *P. coccineus* and *Vigna spp.* (Lavin et al. 2005), which is *μ* = 1.23 × 10–3 substitutions per site per year. The Monte Carlo Markov chain was set to 100,000,000, and two independent runs were combined.

## Results

### Identification and analysis of SNP variation in the chloroplast genome

We selected 70 *P. vulgaris* wild accessions covering the geographical distribution of wild common bean from northern Mexico to northwestern Argentina, thus representing all three gene pools (44 Mesoamerican, 22 Andean and 4 northern Peru/Ecuador, the latter with the ancestral type I phaseolin protein). We also selected wild samples of *P. coccineus* (22) and *P. lunatus* (3), as well as one wild and one domesticated accession of *P. acutifolius*. Across the 97 *Phaseolus* samples, we identified 4008 SNPs (including 777 singletons) 1999 of which were found within genes. Among the 128 chloroplast genes, 66% (84) contained at least one SNP and 45% (56) contained more than three. The most variable genes were ndhF, accD and the pseudogene ycf1b, with 100, 115 and 391 SNPs, respectively. We found 1526 SNPs within exons and 473 within introns, and in the psbH gene all variants were located only within exons. Most of the SNPs were distributed across the single copy regions. A higher SNP density was found in the small single copy region (522 SNPs per 100-bp window on average) compared to the large single copy region (376 SNPs per 100-bp window on average). We found that 42.3% of the variants were synonymous and 57.7% were non-synonymous, with 56.97% of the non-synonymous variants resulting in missense mutations.

### Chloroplast genetic structure

**Fig. 1:**
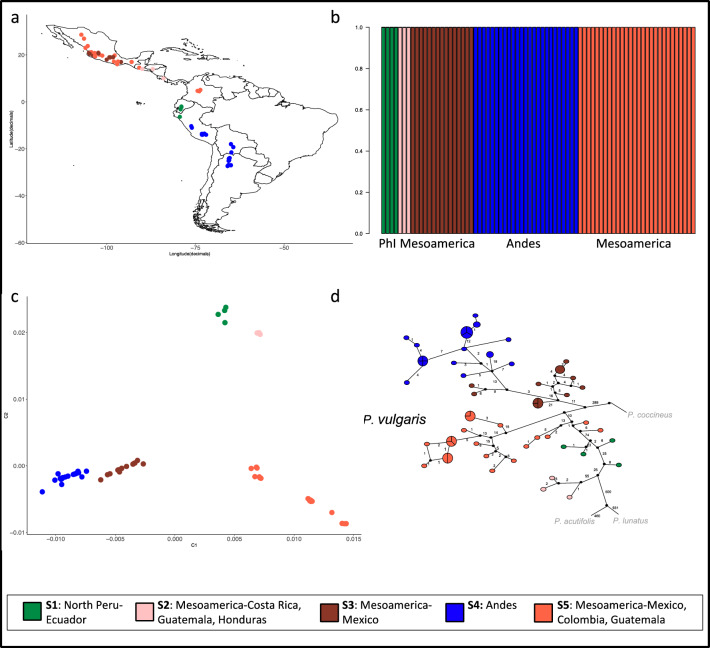
Analysis of the *P. vulgaris* population structure. **a** Geographical distribution of *P. vulgaris* accessions based on BAPS cluster membership. **b** Population structure. **c** MDS plot of *P. vulgaris* samples. **d** Haplotype network analysis of all *Phaseolus* accessions, focusing on *P. vulgaris*. *MW* Mesoamerican wild; *AW* Andean wild; *PhI* Phaseolin type I. Each circle represents a single haplotype, and the size of the circles is proportional to the number of individuals carrying the same haplotype. Black dots indicate missing intermediate haplotypes, and numbers correspond to mutational steps

Characteristics of chloroplast DNA such as uniparental inheritance, haploidy and lack of recombination make it more suitable than nuclear DNA for the reconstruction of intraspecific phylogenetic relationships. We analyzed the chloroplast diversity of a large sample of ~ 100 wild *Phaseolus* accessions, specifically *n* = 70 *P. vulgaris*, *n* = 22 *P. coccineus*, *n* = 2 *P. acutifolius* and *n* = 3 *P. lunatus* (Supplementary Table 1). We also prepared de novo assemblies of 37 aligned chloroplast genomes (Supplementary Figs. 1–4) and used them to corroborate the results obtained from artificial sequences prepared by the concatenation of SNPs. We used 3231 SNPs to investigate the genetic structure of the *P. vulgaris* chloroplast (Fig. 1a,b). The whole population can be divided into five subgroups named S1–S5 (Fig. 1b) based on a log marginal likelihood of optimal partition of –6689.6434. All PhI samples in the panel were pooled in S1. The Mesoamerican accessions were split across S2, S3 and S5. Cluster S2 included three samples from Guatemala, Costa Rica and Honduras, whereas cluster S3 only included Mexican accessions and cluster S5 contained 25 samples from Mexico, two from Colombia and one from Guatemala. All Andean accessions were clustered in S4. Relationships between and within species were inspected by multidimensional scaling (MDS) based on an identity-by-state matrix used as a distance matrix. The MDS plot separated the 97 accessions by species (Supplementary Fig. 5). Specifically, the first component (C1) separated *P. coccineus*, *P. acutifolius* and *P. lunatus*, whereas the second (C2) separated *P. vulgaris* and *P. coccineus*. Within the *P. vulgaris* group (Fig. 1c), C1 separated the Andean and Mesoamerican gene pools and also divided the accessions belonging to the latter into three groups, one of which was closest to the Andean samples. C2 separated the PhI gene pool from the Andean and Mesoamerican gene pools and also separated accessions 59_Pv_MW_CR, 787a_Pv_MW_GT and 790_MW_HN from the rest of the Mesoamerican samples.

Haplotype network analysis was used to visualize pedigree relationships at the intraspecific level. Forty-five haplotypes were identified within the species *P. vulgaris* (Fig. 1d), and no haplotypes were shared between the Mesoamerican and Andean gene pools. Within the Mesoamerican gene pool, four Mexican and three Columbian accessions shared the same haplotype, as did two samples from Mexico and one from Guatemala. Furthermore, in the Andean gene pool, two samples from Peru shared the haplotype with an accession from Bolivia and seven samples from Argentina. The accessions carrying phaseolin type I showed haplotypes that were mostly separated from the Andean samples and closest to the Mesoamerican gene pool, particularly to the three accessions from Guatemala, Honduras and Costa Rica (787a_Pv_MW_GT, 790_Pv_MW_HN and 059_Pv_MW_CR, respectively).

### Phylogenetic analysis of chloroplast DNA

**Fig. 2:**
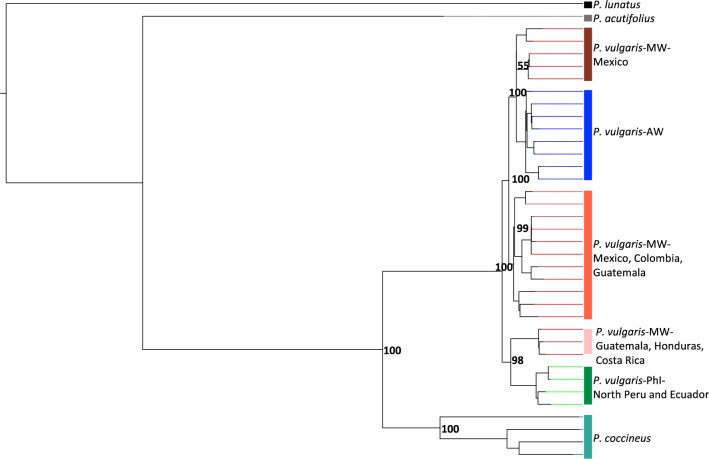
Maximum-likelihood tree obtained from the alignment of 37 chloroplast genomes (bootstrap value = 10,000). *MW* Mesoamerican wild; *AW* Andean wild; *PhI* Phaseolin type I (northern Peru/Ecuador)

We investigated the phylogenetic relationships among the *P. vulgaris* accessions based on the alignment of the 37 de novo chloroplast genomes (Fig. 2). The maximum-likelihood (ML) tree clearly showed the presence of a genetic structure in Mesoamerica with three well supported groups (bootstrap values > 70%). The first included accessions from Guatemala, Honduras and Costa Rica and was more closely related to the northern Peru/Ecuador gene pool. The second included only Mexican accessions pooled with Andean samples, and the third consisted of samples from Mexico, Guatemala and Colombia. The clade of accessions from northern Peru/Ecuador clearly arose from the Mesoamerican gene pool. The ML tree constructed from SNP data (Supplementary Fig. 6) were topologically consistent with the tree constructed from the chloroplast genomes.

### Molecular clock analysis of chloroplast DNA

**Fig. 3:**
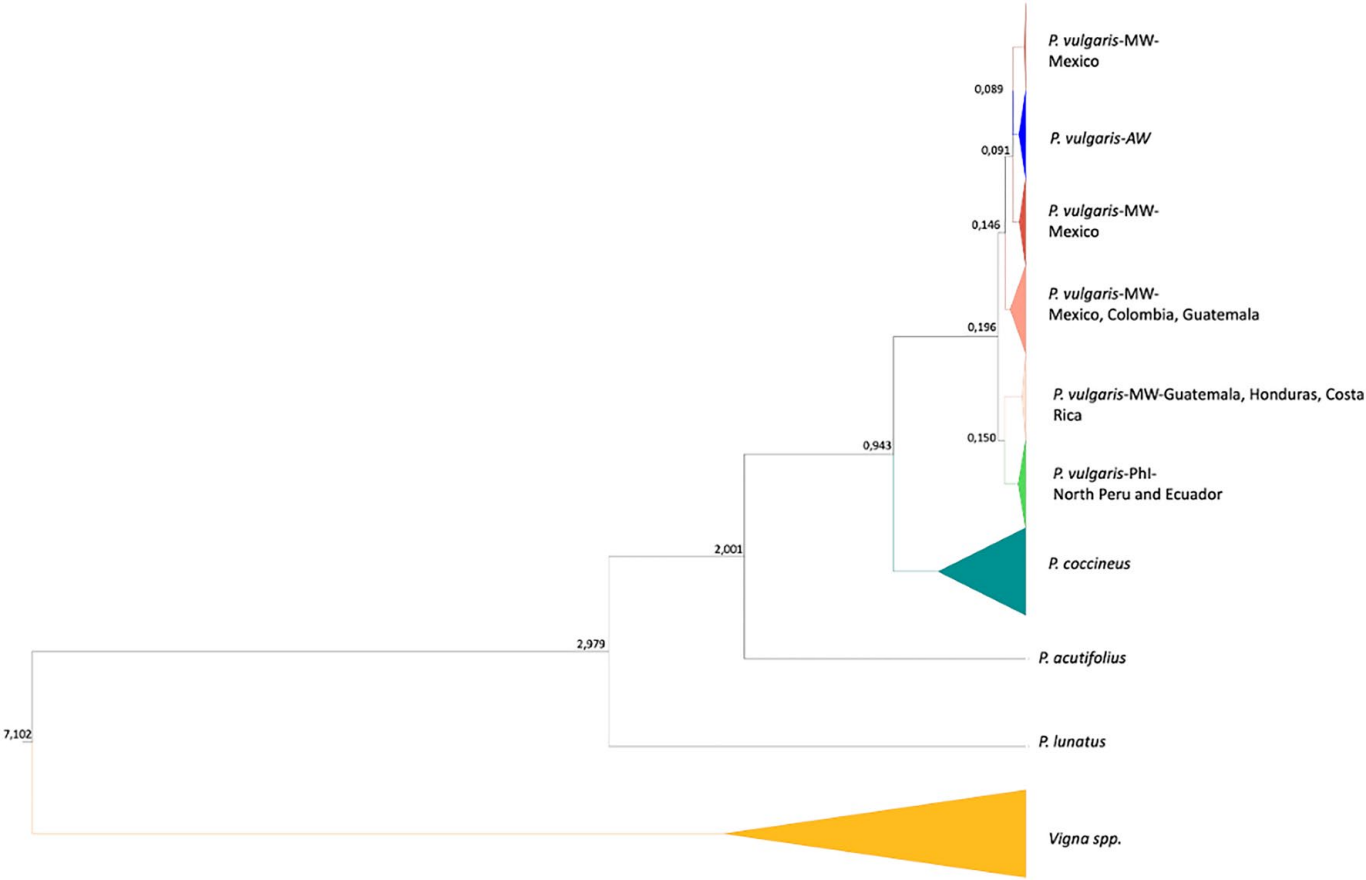
Molecular clock analysis computed using 37 chloroplast genomes. Divergence times are shown on the nodes. *MW* Mesoamerican wild; *AW* Andean wild; *PhI* Phaseolin type I (northern Peru/Ecuador)

To estimate timelines for the divergence of the *P. vulgaris* gene pools, we combined the 37 de novo chloroplast genome assemblies with three *Vigna spp.* plastomes and carried out a coalescent simulation with a relaxed log-normal molecular clock, using the divergence between *P. coccineus* and *Vigna spp.* to calibrate the tree (Lavin et al. 2005). The coalescent simulation (Fig. 3) showed a divergence time of ~ 0.19 million years ago (Mya) between the wild *P. vulgaris* genetic groups (0.0847–0.3082 95% highest posterior probability, HPD). The separation between the northern Peru/Ecuador gene pool and the group comprising the Mesoamerican and Andean gene pools occurred ~ 0.15 Mya (0.0607–0.2419 95% HPD). A split between the Mesoamerican and Andean populations was very recent, at ~ 0.09 Mya (0.0422–0.1515 95% HPD).

### Intraspecies phylogenetic analysis of the nuclear genome

Nuclear data from 10 *P. vulgaris* accessions representing the three wild gene pools (Supplementary Table 2) were also analyzed to infer phylogenetic relationships. We identified 11,160,422 SNPs, most of which were found in intergenic (45.30%), upstream (19.88%) or downstream (20.00%) regions. A further 7.45% of the SNPs were found within introns and 4.86% within exons. Only SNPs located in the pericentromeric regions, which are less prone to recombination, were used to construct an ML tree for each of the 11 chromosomes (Supplementary Table 3).

**Fig. 4:**
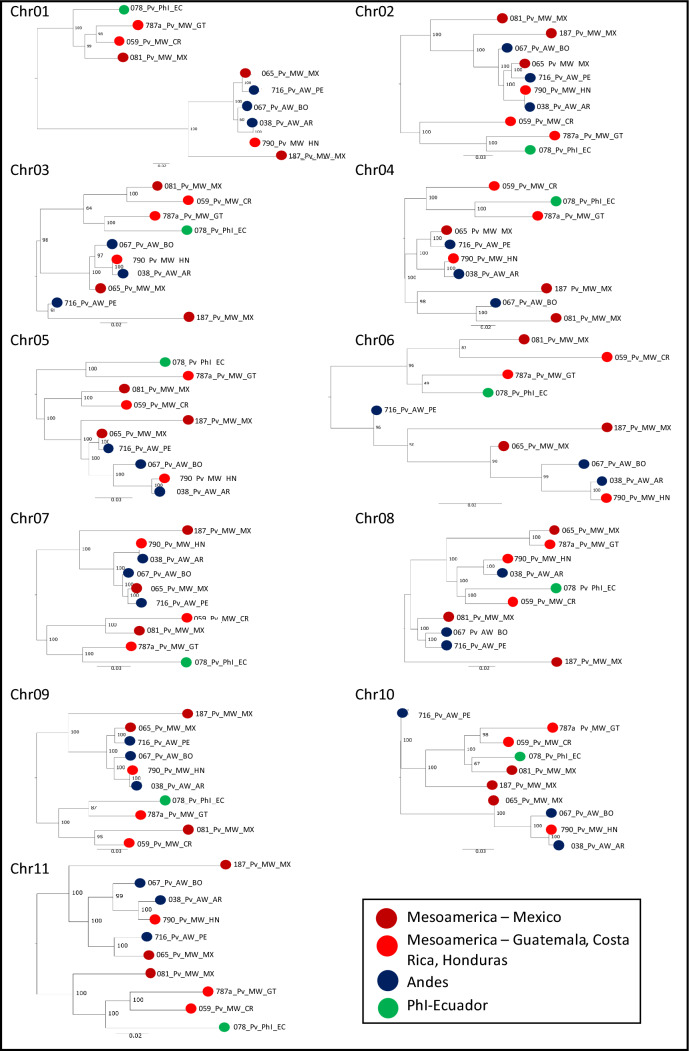
Maximum-likelihood trees based on concatenated SNPs extracted from the pericentromeric regions of the 11 chromosomes. Dark red: Mesoamerican samples from Mexico. Light red: Mesoamerican samples from Guatemala, Costa Rica or Honduras. Blue: Andean samples. Green: PhI sample from Ecuador

Although different topologies were obtained for the 11 chromosome-specific ML trees (Fig. 4), most nodes were well supported (bootstrap values > 70%). No clear distinction was observed between the Mesoamerican and Andean gene pools. Interestingly, PhI and Mesoamerican samples from Costa Rica, Guatemala and Mexico (Oaxaca) grouped together (078_Pv_PhI_EC, 059_Pv_MW_CR, 787a_Pv_MW_GT and 081_Pv_MW_MX). We also constructed a neighbor-joining tree from concatemers of genome-wide SNPs pruned every 250 kb (Supplementary Fig. 7). Contrary to the results obtained with chloroplast sequences, the nuclear data were insufficient to make inferences about the origin of and divergence between the three wild gene pools.

## Discussion

Knowledge of the origin, evolution and diffusion of crops is necessary for the appropriate use and conservation of available genetic resources. Wild forms are characterized by extensive genetic and phenotypic diversity, which firstly must be recovered and exploited to accelerate breeding programs (Gepts 1990) and secondly, its study allows to trace back the evolutionary history of a species.

Indeed, the reconstruction of a reliable phylogeny is hard when domesticated forms are considered due to their genetic flow with the wild forms, the admixture between different cultigens together with the reduction of the genetic variability caused by the domestication process itself.

In this study, we carried out an intraspecific phylogenetic analysis based on chloroplast and nuclear sequence data to elucidate the relationships among wild *Phaseolus vulgaris* gene pools. We analyzed the genetic diversity of a large collection of *P. vulgaris* chloroplast genomes representing the three wild gene pools. Compared with previous work (Rendón-Anaya et al. 2017b), this gave us the possibility to consider the issue of the PhI origin within a high-resolution picture of the chloroplast genetic diversity of wild *P. vulgaris*. A selection of these genotypes was then used to identify nuclear SNPs suitable to reconstruct the phylogeography of the common bean and clarify the relationships and timeframe for the divergence of the wild genetic groups.

Our results assigned PhI accessions from northern Peru/Ecuador to a clade that clearly arose from the Mesoamerican gene pool, in agreement with earlier reports (Bitocchi et al. 2012). This was supported by complementary phylogenesis, Bayesian analysis of population structure (BAPS), MDS and haplotype network analysis, revealing a clear subdivision of the Mesoamerican population. Two of the Mesoamerican subpopulations were placed closest to the Andean gene pool and PhI accessions, respectively. Similar results were reported by Bitocchi et al. (2012) and Desiderio et al. (2013) using different molecular markers. Our findings therefore support the monophyletic and Mesoamerican origin of the common bean and revealed no evidence supporting PhI population speciation, as proposed by (Rendón-Anaya et al. 2017a, b). The estimated divergence times between *P. vulgaris* gene pools provided additional supporting evidence. The coalescent simulation showed a divergence time between wild *P. vulgaris* groups of ~ 0.19 Mya.

Our estimate of ~ 0.19 Mya overlaps with that computed by Rendón-Anaya et al. (2017b) for the split between a domesticated Andean genotype and a domesticated Mesoamerican genotype but differs greatly compared to the split between a wild genotype (G21245) from northern Peru/Ecuador and the aforementioned Andean and Mesoamerica genotypes (0.9 Mya). Based on our data, the divergence between the Mesoamerican and Andean gene pools occurred much earlier ~ 0.09 Mya, which is similar to earlier estimates of ~ 0.11 Mya (Mamidi et al. 2013) and ~ 0.087 Mya (Ariani et al. 2018). The different outcomes of the present work compared to the results obtained by Rendón-Anaya et al. (2017b) may be explained by the different sampling strategies. Indeed, we selected a large sample (70) of wild accessions of common bean contrary to the other authors that estimated the divergence times with domesticated samples using only two domesticated sample from Mesoamerica and Andes (BAT93 and Jalo EEP558).

Based on all lines of evidence, we propose that two migratory events occurred, and both originated in Mesoamerica. The first migratory event allowed the common bean to spread to northern Peru/Ecuador about 150,000 years ago. A more recent migration then gave rise the Andean gene pool ~ 90,000 years ago. Indeed, a single migration event would imply an initial adaptation to the equatorial environment and a subsequent adaptation to the negative latitude and high altitude. Furthermore, based on the same rationale, we suggest that the migration from Mesoamerica (Costa Rica) to northern Peru/Ecuador was facilitated by migratory birds flying over the Pacific Ocean. The second migration could involve either of two main bird migration routes from North to South America (Zimmer 1938), one through the Gulf of Mexico and a longer one through Central America passing across the neck of Panama and then following the course of the Andes, or the west coast, or the northern coast eastward from Panama, or a diagonal course southeast through the Amazonian region. Migratory birds may therefore be responsible for several long-distance dispersal events (Remsen 1984; La Sorte et al. 2016; Viana et al. 2016) that led to the current distribution of the wild common bean. Differently, Ariani et al. (2018) proposed at least three long-distance migratory events from the center of origin in Mexico to southern Mesoamerica, northern Peru/Ecuador and the southern Andes. However, our results indicate the occurrence of just two migration events (Ariani et al. 2018). The higher loss of nucleotide diversity in the Andean gene pool compared to the Mesoamerican one suggests that concurrently with the migration events other evolutionary forces such as drift and natural selection might have acted on the common bean gene pools resulting in a strong differentiation of the three genetic groups. In addition to the higher loss of nucleotide diversity found in the wild Andean gene pool compared to the Mesoamerican one (Rossi et al. 2009; Bitocchi et al. 2012; Desiderio et al. 2013; Ariani et al. 2018), the recent study conducted on the common bean pangenome revealed the progressive loss of genes in wild genotypes ranging from Northern Mexico to Northwestern Argentina, indicating a potential adaptive role during wild range expansion (Cortinovis et al. 2024).

Our attempt to reconstruct the phylogenetic history of the common bean using nuclear markers provides a clear example of the bias introduced by the use of markers from DNA regions subject to recombination. Indeed, one of the main assumptions during phylogenetic reconstruction is the absence of recombination. Ignoring crossovers, gene conversion, horizontal transfer and hybridization can lead to erroneous estimates that do not represent any of the probable evolutionary histories of the species.

Previous studies in which phylogenesis was carried out using genome-wide markers placed PhI samples on the outermost branches (Papa and Gepts 2003; Rendón-Anaya et al. 2017a, b). To overcome the consequences of recombination and especially crossover events, we restricted our analysis to SNPs located in centromeric regions, which are cold spots for recombination (Lischer and Excoffier 2012). However, the analysis of centromeric markers did not provide sufficient resolution to infer intraspecific phylogenetic relationships among *P. vulgaris* genotypes. Indeed, it was not possible to make, from different chromosomes, unique inferences about the derivation of PhI. Introgression, incomplete lineage sorting and gene conversion may have acted as non-reciprocal recombination events even in the absence of crossovers (Talbert and Henikoff 2010), making the phylogenetic approach based on nuclear markers unsuitable for detecting close relationships, such as those between gene pools of the same species. Nevertheless, the 11 trees (one for each centromeric region) revealed that even though the centromeres had slightly different topologies, the PhI sample visibly showed a relationship to the Mesoamerican gene pool and specifically with accessions from Guatemala, Costa Rica and the valley of Oaxaca, the latter suggested as the presumed center of domestication (Bitocchi et al. 2013; Rodriguez et al. 2016). The proximity of the PhI accession to the Mesoamerican gene pool was also observed in the analysis of the core SNPs detected using the common bean pangenome (Cortinovis et al. 2024).

Previous studies in which phylogenesis was carried out using genome-wide markers placed PhI samples on the outermost branches (Papa and Gepts 2003; Rendón-Anaya et al. 2017a, b). To overcome the consequences of recombination and especially crossover events, we restricted our analysis to SNPs located in centromeric regions, which are cold spots for recombination (Lischer and Excoffier 2012). However, the analysis of centromeric markers did not provide sufficient resolution to infer intraspecific phylogenetic relationships among *P. vulgaris* genotypes. Indeed, it was not possible to make, from different chromosomes, unique inferences about the derivation of PhI. Introgression, incomplete lineage sorting and gene conversion may have acted as non-reciprocal recombination events even in the absence of crossovers (Talbert and Henikoff 2010), making the phylogenetic approach based on nuclear markers unsuitable for detecting close relationships, such as those between gene pools of the same species. Nevertheless, the 11 trees (one for each centromeric region) revealed that even though the centromeres had slightly different topologies, the PhI sample visibly showed a relationship to the Mesoamerican gene pool and specifically with accessions from Guatemala, Costa Rica and the valley of Oaxaca, the latter suggested as the presumed center of domestication (Bitocchi et al. 2013; Rodriguez et al. 2016). The increasing availability of pangenomes and the development of pangenomic approaches for phylogenetic studies already revealed the complex phylogenetic relationships among bacterial strains (Yang et al. 2023), it is desirable that the advancements in the efficiency of pangenomic tools will soon enable the resolution of the phylogeny of eucaryotic organisms as well. Indeed, graph pangenomes can potentially capture all type of genomic variation allowing the evaluation of highly recombinant populations (Secomandi et al. 2025).

Our work is an example of phylogenetics applied to the evolutionary history of populations belonging to the same biological species and in particular the three wild gene pools of *P. vulgaris*. Despite the limited number of PhI accessionss, our findings confirm the monophyletic and Mesoamerican origin of the common bean but also provide a deeper understanding of the relationships between the three major wild gen pools and their divergence events. In addition, we provide clear evidence of bias due to recombination events when using nuclear data to reconstruct phylogenetic trees. Our study therefore clarifies the intraspecific phylogeny of *P. vulgaris* and its origin in Mesoamerica. Further studies conducted on high-resolution pangenomes might help overcoming the low resolution of phylogenetic studies employing nuclear genome data. *P. vulgaris* can be considered as a unique model for the study of crop evolution due to both the presence of three distinct wild gene pools and the occurrence of two domestication events (Bitocchi et al. 2012, 2013). Therefore, the understanding of the relationships among its wild gene pools not only is essential for the adequate conservation of common bean genetic resources but it is also relevant for other legume species and specifically for those closely related such as *P. domusus* and *P. coccineus*.

## Supplementary Information

Below is the link to the electronic supplementary material.

Supplementary file1 (PDF 2418 KB)

## Data Availability

The raw sequence reads generated and analyzed during this study are available in the Sequence Read Archive (SRA) of the National Center for Biotechnology Information (NCBI) with the BioProject number PRJNA910538. The bioinformatics pipelines used in this study are available in https://github.com/giuliafrascarelli/Common_bean.
