# Supplementary material for: The evolutionary history of the common bean (Phaseolus vulgaris) revealed by chloroplast and nuclear genomes analysis
Source: Theor Appl Genet. 2025 Feb 8;138(2):47. doi: 10.1007/s00122-025-04832-z (PMC11805837; doi:10.1007/s00122-025-04832-z)
Supplement: Supplementary file 1 — Supplementary file1 (PDF 2418 KB) [file 122_2025_4832_MOESM1_ESM.pdf]

Supplementary Materials

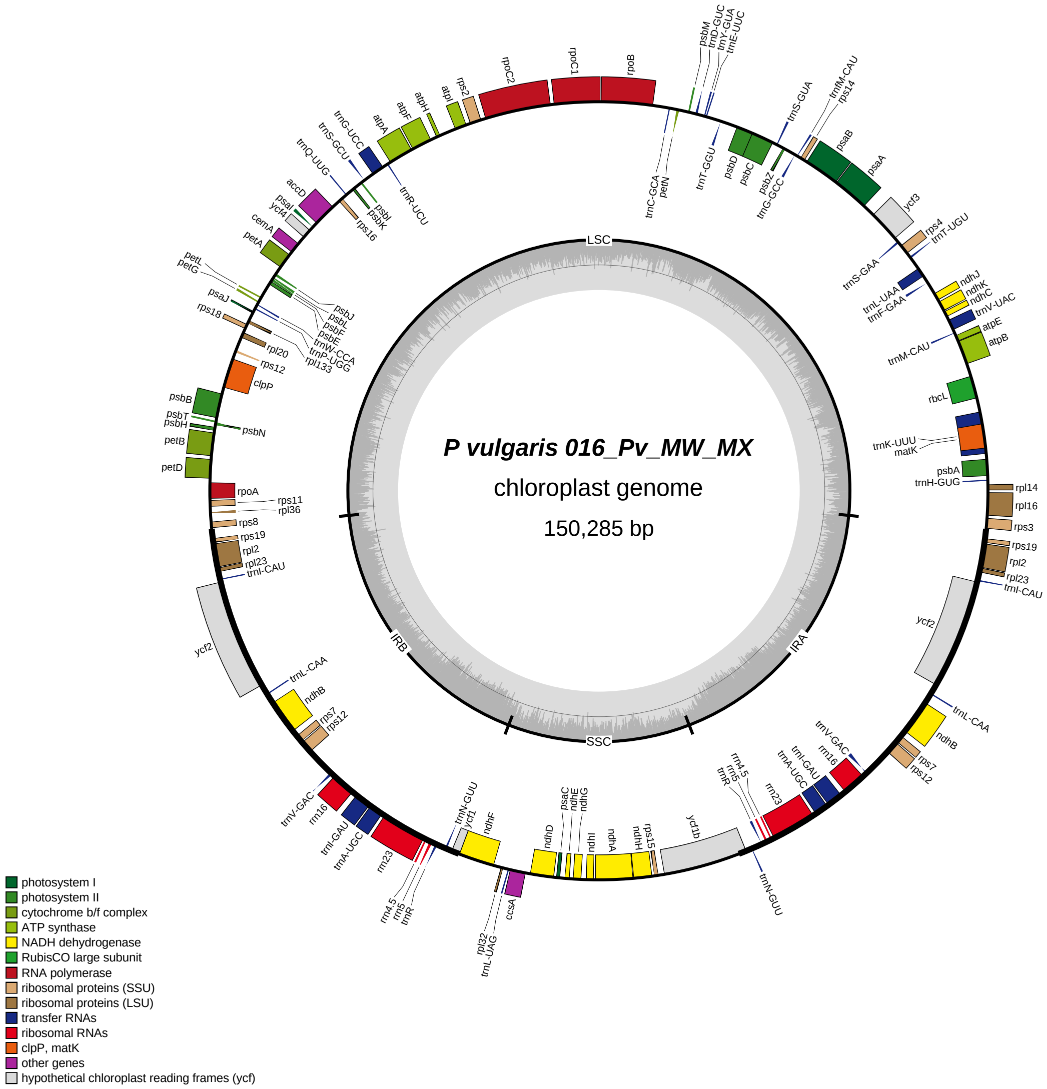

Supplementary_Figure_1: Map of the chloroplast genome of the accessions 016_Pv_MW_MX (*Phaseolus vulgaris*). Genes inside of the outer circle are transcribed in the clockwise direction, while those outsides are transcribed in the counterclockwise direction. Different color codes represent genes belonging to various functional groups. The circle inside represents GC content graph with the 50% threshold. The inverted repeat, large single-copy, and small single-copy regions are denoted by IR, LSC, and SSC, respectively.

Comparative analysis

To visualize the differences among the assembled chloroplast genomes, a multiple alignment was built in mVISTA (Stanford University, Stanford CA, USA) in LAGAN (Limited Area Global Alignment of Nucleotides) mode using *P. vulgaris* published plastome and its annotation as reference (NC_009259). An additional alignment was carried out using the MAUVE alignment software (Darling et al. 2004). This alignment was performed on a subset of plastomes, chosen based on the gene content differences revealed by the annotation, using the progressive MAUVE option.

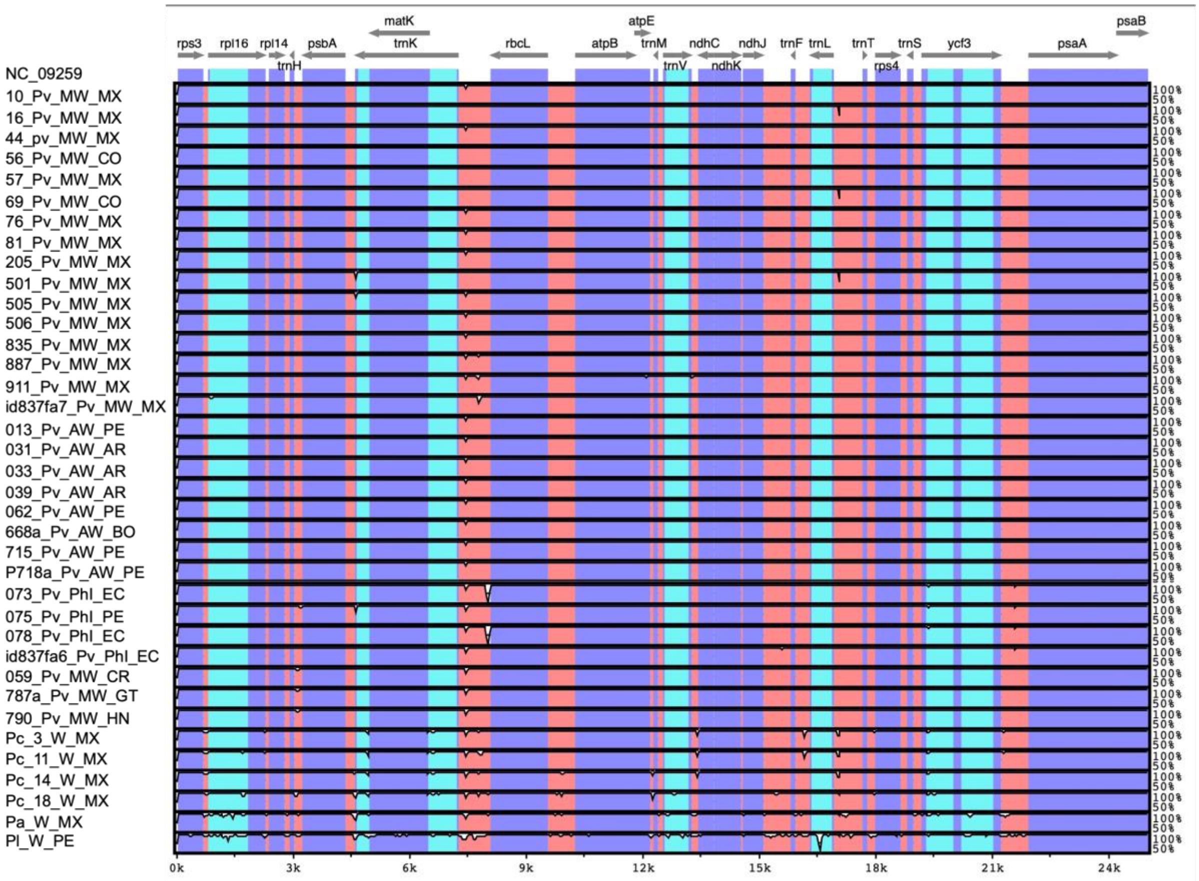

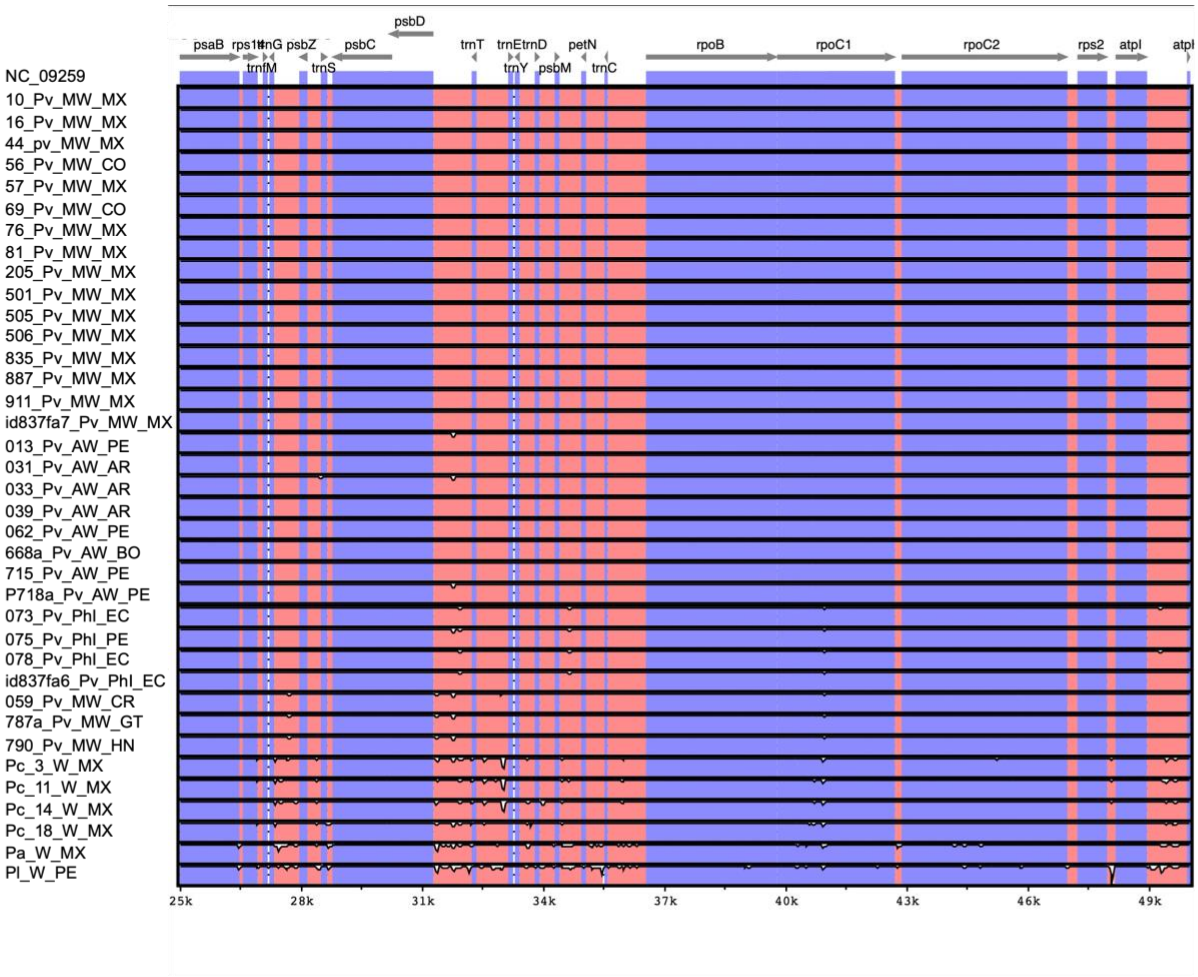
psbD
psaB
rps14
trnG
psbZ
psbC
trnfM
trnS
trnT
trnE
trnD
trnY
psbM
petN
trnC
rpoB
rpoC1
rpoC2
rps2
atpI
NC_09259
10_Pv_MW_MX
16_Pv_MW_MX
44_pv_MW_MX
56_Pv_MW_CO
57_Pv_MW_MX
69_Pv_MW_CO
76_Pv_MW_MX
81_Pv_MW_MX
205_Pv_MW_MX
501_Pv_MW_MX
505_Pv_MW_MX
506_Pv_MW_MX
835_Pv_MW_MX
887_Pv_MW_MX
911_Pv_MW_MX
id837fa7_Pv_MW_MX
013_Pv_AW_PE
031_Pv_AW_AR
033_Pv_AW_AR
039_Pv_AW_AR
062_Pv_AW_PE
668a_Pv_AW_BO
715_Pv_AW_PE
P718a_Pv_AW_PE
073_Pv_Phl_EC
075_Pv_Phl_PE
078_Pv_Phl_EC
id837fa6_Pv_Phl_EC
059_Pv_MW_CR
787a_Pv_MW_GT
790_Pv_MW_HN
Pc_3_W_MX
Pc_11_W_MX
Pc_14_W_MX
Pc_18_W_MX
Pa_W_MX
Pl_W_PE
25k
28k
31k
34k
37k
40k
43k
46k
49k

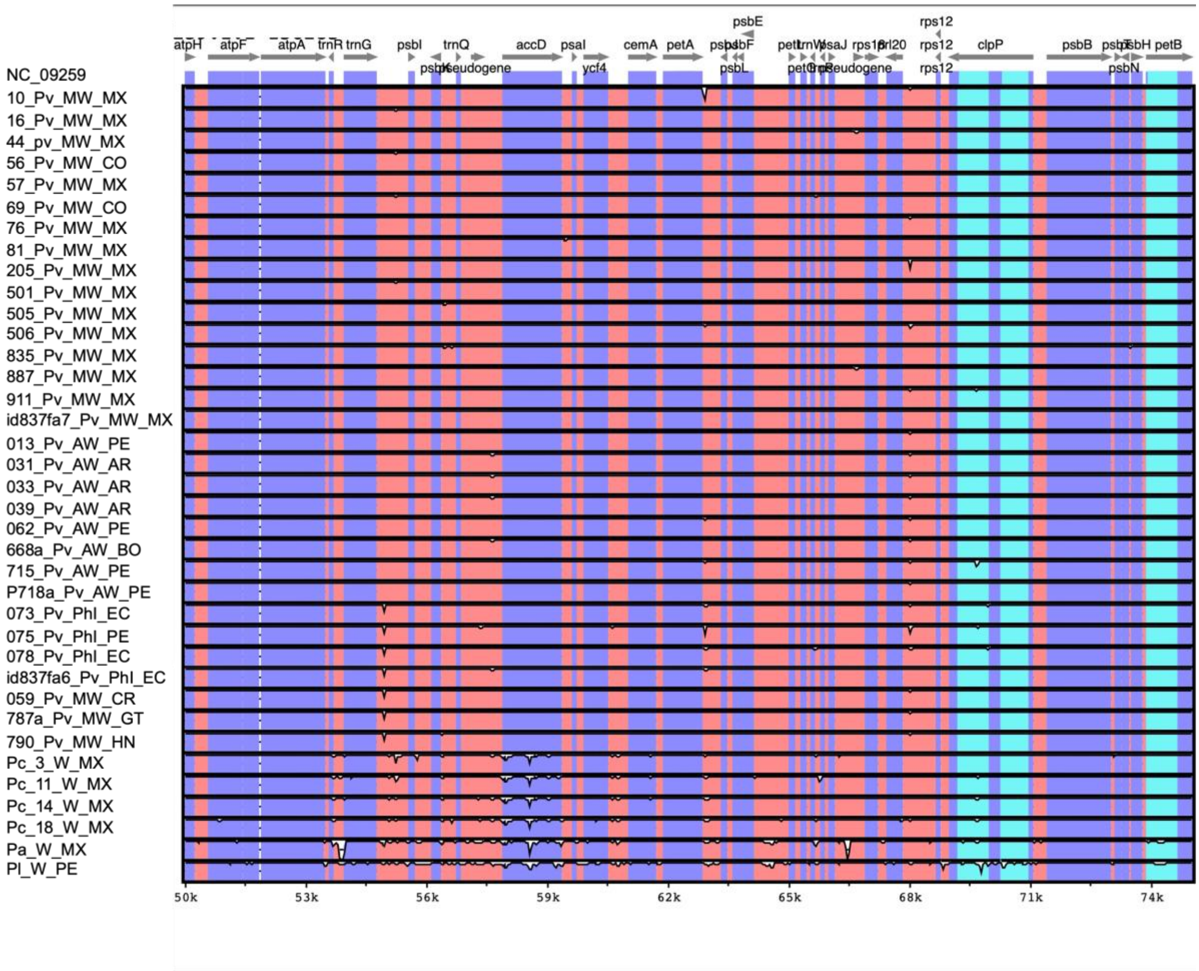
psbE
rps12
atpH
atpF
atpA
trnR
trnG
psbI
trnQ
accD
psaI
cemA
petA
petL
rps12
clpP
psbB
psbH
petB
ycf4
psbL
psbN
NC_09259
10_Pv_MW_MX
16_Pv_MW_MX
44_pv_MW_MX
56_Pv_MW_CO
57_Pv_MW_MX
69_Pv_MW_CO
76_Pv_MW_MX
81_Pv_MW_MX
205_Pv_MW_MX
501_Pv_MW_MX
505_Pv_MW_MX
506_Pv_MW_MX
835_Pv_MW_MX
887_Pv_MW_MX
911_Pv_MW_MX
id837fa7_Pv_MW_MX
013_Pv_AW_PE
031_Pv_AW_AR
033_Pv_AW_AR
039_Pv_AW_AR
062_Pv_AW_PE
668a_Pv_AW_BO
715_Pv_AW_PE
P718a_Pv_AW_PE
073_Pv_PhI_EC
075_Pv_PhI_PE
078_Pv_PhI_EC
id837fa6_Pv_PhI_EC
059_Pv_MW_CR
787a_Pv_MW_GT
790_Pv_MW_HN
Pc_3_W_MX
Pc_11_W_MX
Pc_14_W_MX
Pc_18_W_MX
Pa_W_MX
Pl_W_PE
50k
53k
56k
59k
62k
65k
68k
71k
74k

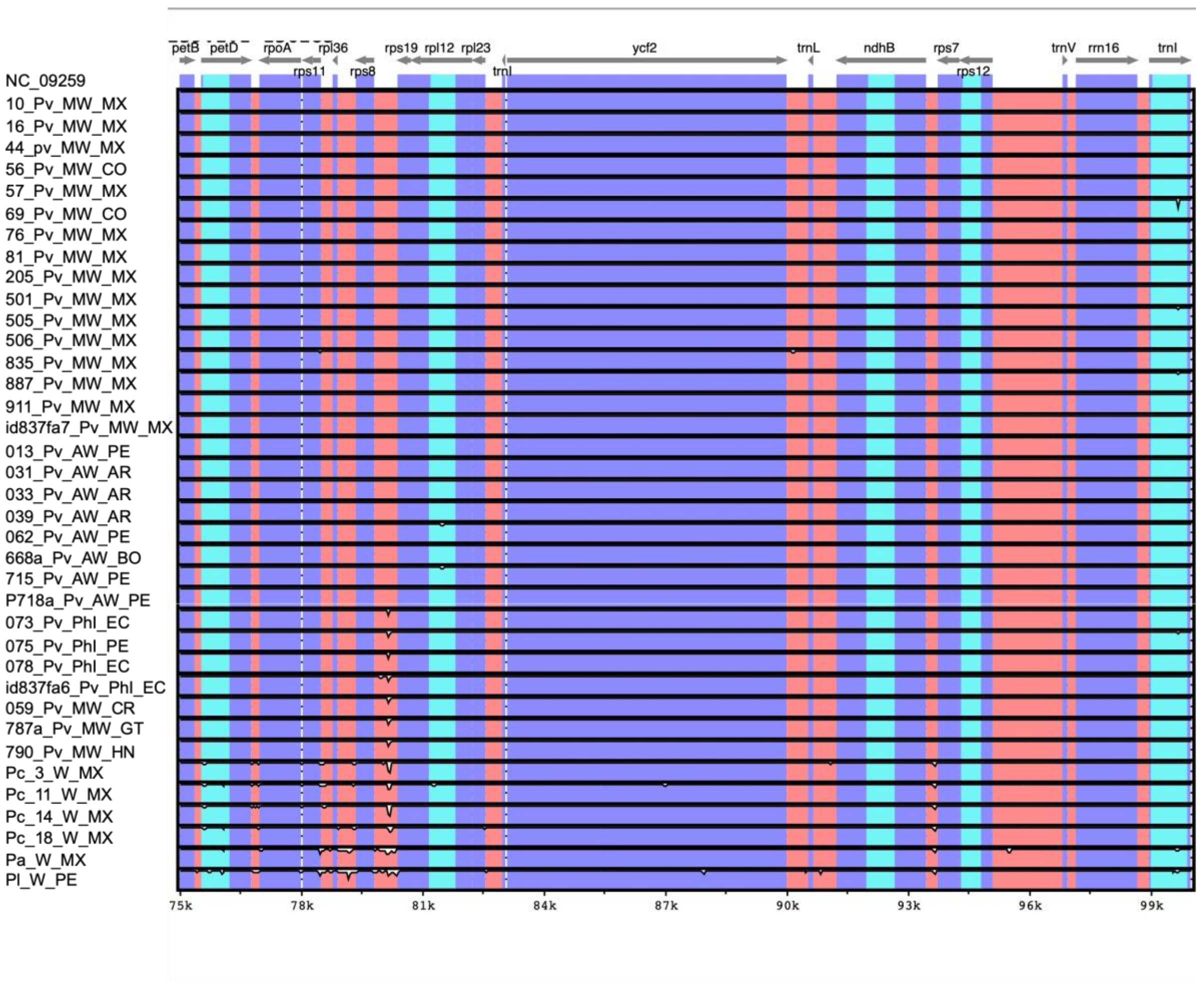
petB
petD
rpoA
rpl36
rps11
rps8
rps19
rpl12
rpl23
trnI
ycf2
trnL
ndhB
rps7
rps12
trnV
rrn16
trnI
NC_09259
10_Pv_MW_MX
16_Pv_MW_MX
44_pv_MW_MX
56_Pv_MW_CO
57_Pv_MW_MX
69_Pv_MW_CO
76_Pv_MW_MX
81_Pv_MW_MX
205_Pv_MW_MX
501_Pv_MW_MX
505_Pv_MW_MX
506_Pv_MW_MX
835_Pv_MW_MX
887_Pv_MW_MX
911_Pv_MW_MX
id837fa7_Pv_MW_MX
013_Pv_AW_PE
031_Pv_AW_AR
033_Pv_AW_AR
039_Pv_AW_AR
062_Pv_AW_PE
668a_Pv_AW_BO
715_Pv_AW_PE
P718a_Pv_AW_PE
073_Pv_Phl_EC
075_Pv_Phl_PE
078_Pv_Phl_EC
id837fa6_Pv_Phl_EC
059_Pv_MW_CR
787a_Pv_MW_GT
790_Pv_MW_HN
Pc_3_W_MX
Pc_11_W_MX
Pc_14_W_MX
Pc_18_W_MX
Pa_W_MX
Pl_W_PE
75k
78k
81k
84k
87k
90k
93k
96k
99k

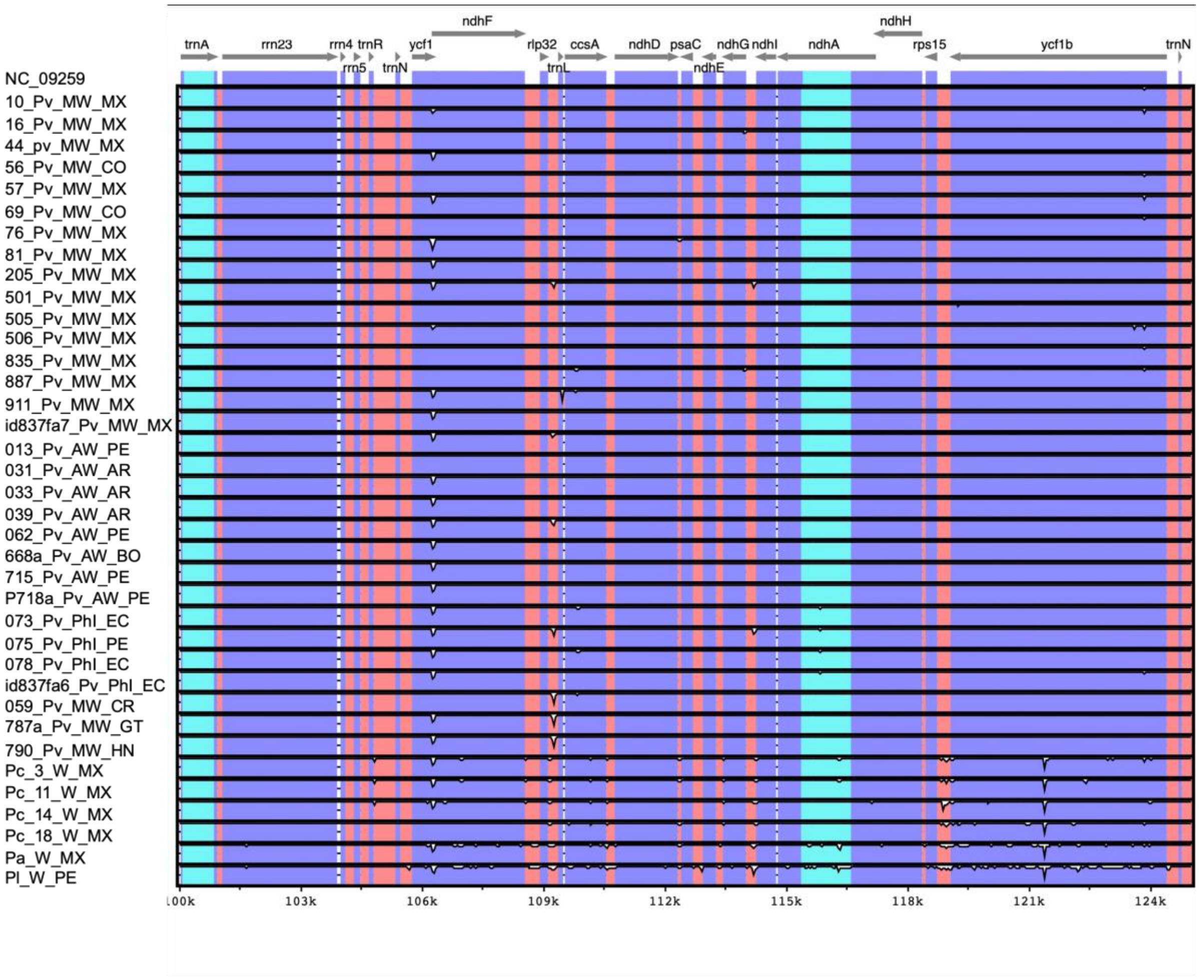

trnA
rrn23
rrn4
trnR
rrn5
trnN
ycf1
ndhF
rlp32
trnL
ccsA
ndhD
psaC
ndhE
ndhG
ndhI
ndhA
ndhH
rps15
ycf1b
trnN
NC_09259
10_Pv_MW_MX
16_Pv_MW_MX
44_pv_MW_MX
56_Pv_MW_CO
57_Pv_MW_MX
69_Pv_MW_CO
76_Pv_MW_MX
81_Pv_MW_MX
205_Pv_MW_MX
501_Pv_MW_MX
505_Pv_MW_MX
506_Pv_MW_MX
835_Pv_MW_MX
887_Pv_MW_MX
911_Pv_MW_MX
id837fa7_Pv_MW_MX
013_Pv_AW_PE
031_Pv_AW_AR
033_Pv_AW_AR
039_Pv_AW_AR
062_Pv_AW_PE
668a_Pv_AW_BO
715_Pv_AW_PE
P718a_Pv_AW_PE
073_Pv_Phl_EC
075_Pv_Phl_PE
078_Pv_Phl_EC
id837fa6_Pv_Phl_EC
059_Pv_MW_CR
787a_Pv_MW_GT
790_Pv_MW_HN
Pc_3_W_MX
Pc_11_W_MX
Pc_14_W_MX
Pc_18_W_MX
Pa_W_MX
Pl_W_PE
100k
103k
106k
109k
112k
115k
118k
121k
124k

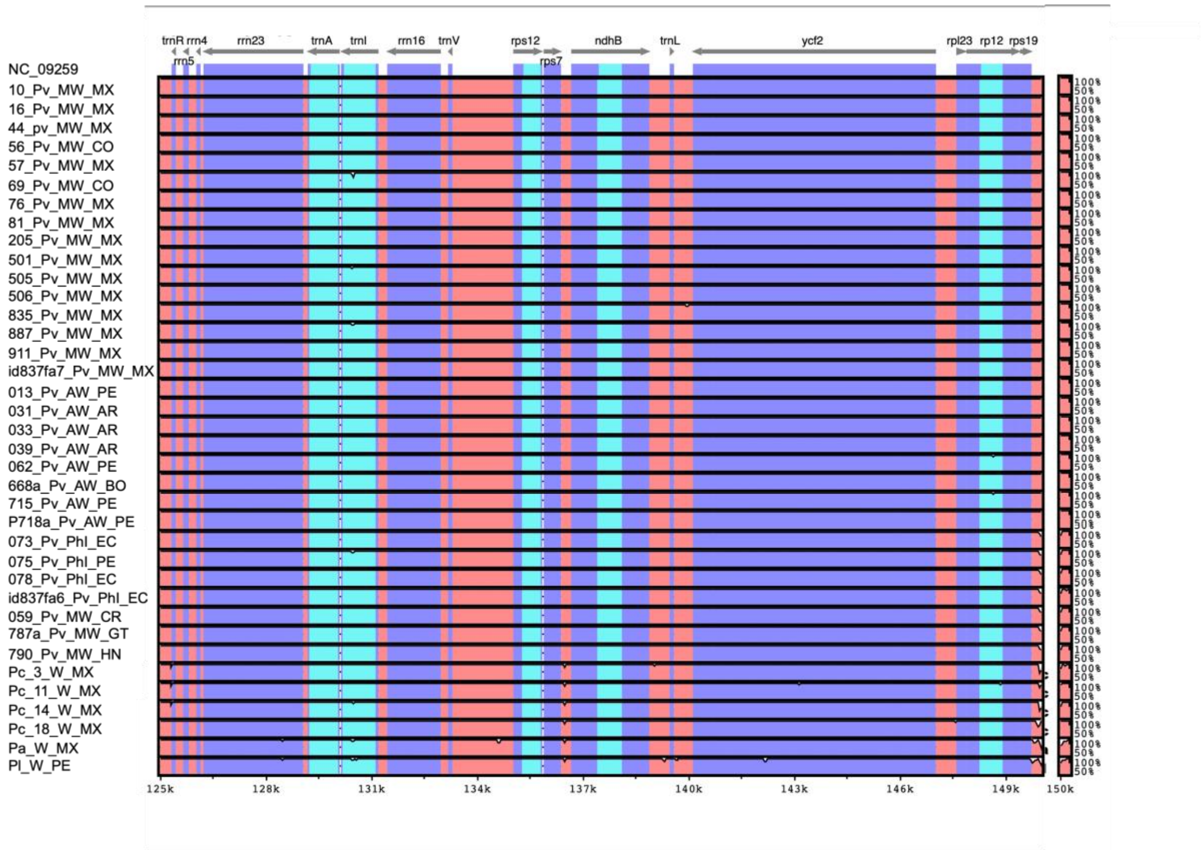

Supplementary_Figure_2: Sequence similarity plot by using mVISTA, among 37 de-novo assembled chloroplast genomes and NC_09259 as reference. In the y-axis percentage of sequence identity was shown between 50% and 100%. Transcriptional orientations of genes were assigned by grey arrows. Red bars represented non-coding sequences (NCS), purples bars represented exons and light blue bars represented untranslated regions (UTRs). Genomic differences were shown as white peaks.

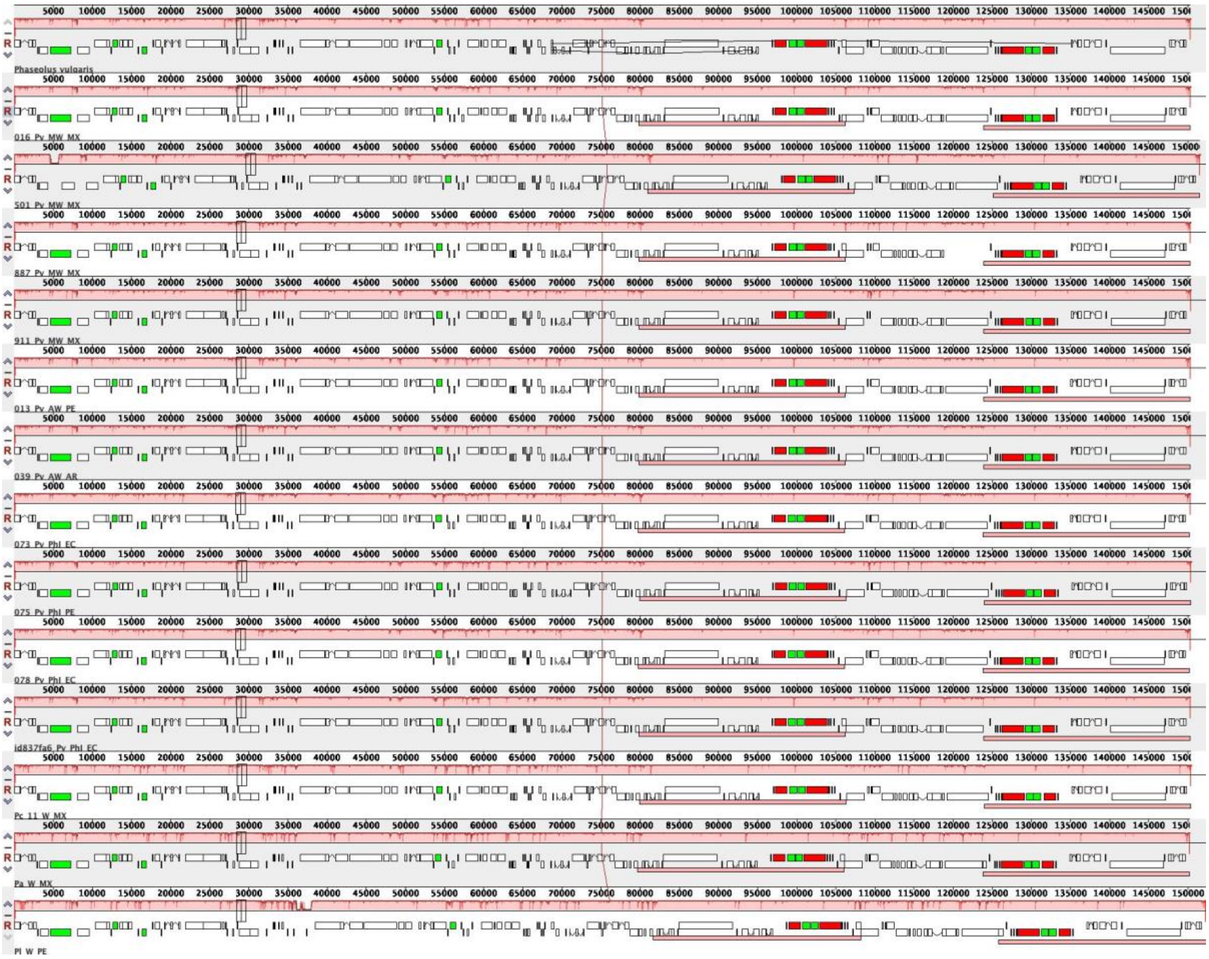

Supplementary_Figure_3: MAUVE alignment showing the gene order and homology between the reference chloroplast genome of *Phaseolus vulgaris* (NC_09259) fifteen *P. vulgari*s accessions (16_Pv_MW_MX, 501_Pv_MW_MX, 887_Pv_MW_MX, 911_Pv_M_MX, 013_Pv_AW_PE0, 39_Pv_AW_AR,54_Pv_PhI_PE, 073_Pv_PhI_EC, 075_Pv_PhI_PE, 078_Pv_PhI_EC), *P. coccineus* (Pc_11_W_MX), *P. acutifolius* (Pa_W_MX), *P. lunatus* (Pl_W_PE). Locally Collinear Blocks (LCBs) include the histograms that show sequence identity with peaks. Protein coding genes, rRNA genes, tRNA genes and intron containing tRNA genes are marked with block in white, red, black and green colors, respectively.

The genome variation among all *de-novo* assembled chloroplast genomes was analyzed with the online tool mVISTA unsing *P. vulgaris* (NC_09259) as reference. The mVISTA identity plot did not reveal meaningful differences between the *P. vulgaris* plastomes, with the exception of a small deletion in the intergenic region between *trnK* and *rbcL* gene, which was found in two PhI samples (i.e., 073_Pv_PhI_EC and 078_Pv_PhI_EC). In addition, a deletion (over 300 pb) was identified in the plastome of the *P. acutifolius* accession (i.e., Pa_W_MX) in the intergenic region between the *trnR* and *trnG* genes (Supprementaly_Figure_2).

To identify the gene order and organization, a subset of 15 plastomes was aligned with MAUVE (Supplementary_Figure_3). Most of the regions were conserved among all plastomes and no rearrangements of gene order was detected.

Analysis of nucleotide diversity

In order to determine nucleotide diversity in the 37 *de-novo* assembled plastomes a multi-sequence alignment (MSA) was performed using MAFFT (Katoh et al., 2019) with default parameters. The MSA was used as input for DNAsp (Rozas et al., 2017) and nucleotide diversity was calculated. A sliding window of 200 bp with 50 bp step size was used to summarize the diversity statistics for visualization.

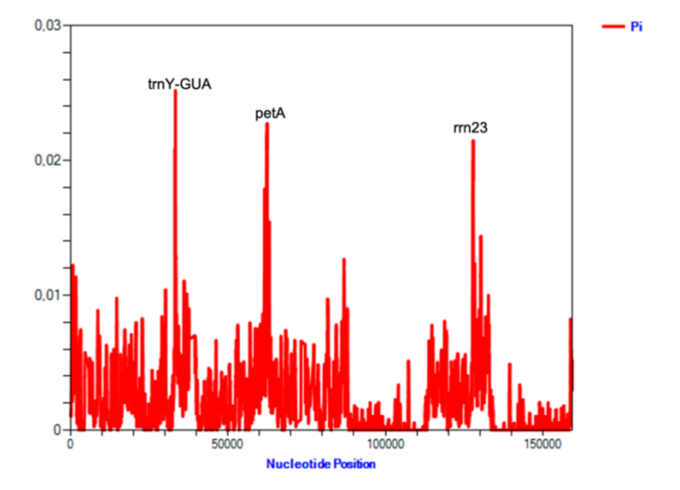

Supplementary_Figure_4: Sliding window analysis among the whole chloroplast genome of 37 de-novo assembled plastomes, including *P. vulgaris, P. coccineus, P. acutifolius, P. lunatus.* Regions with higher nucleotide variability are indicated.

The nucleotide diversity was investigated with DNAsp software. The multi sequence alignment revealed 145133 monomorphic sites 3586 polymorphic sites of which 1254 were defined as informative. A sliding window analysis was performed to calculate the nucleotide variability (Pi) across the 37 *de-novo* chloroplast genomes. Even though, the results showed a high sequence similarity, three divergent hot spots were detected (Pi>0.02): *trnY-GUA*, *petA*, *rrn23* (Supplementary_Figure_4).

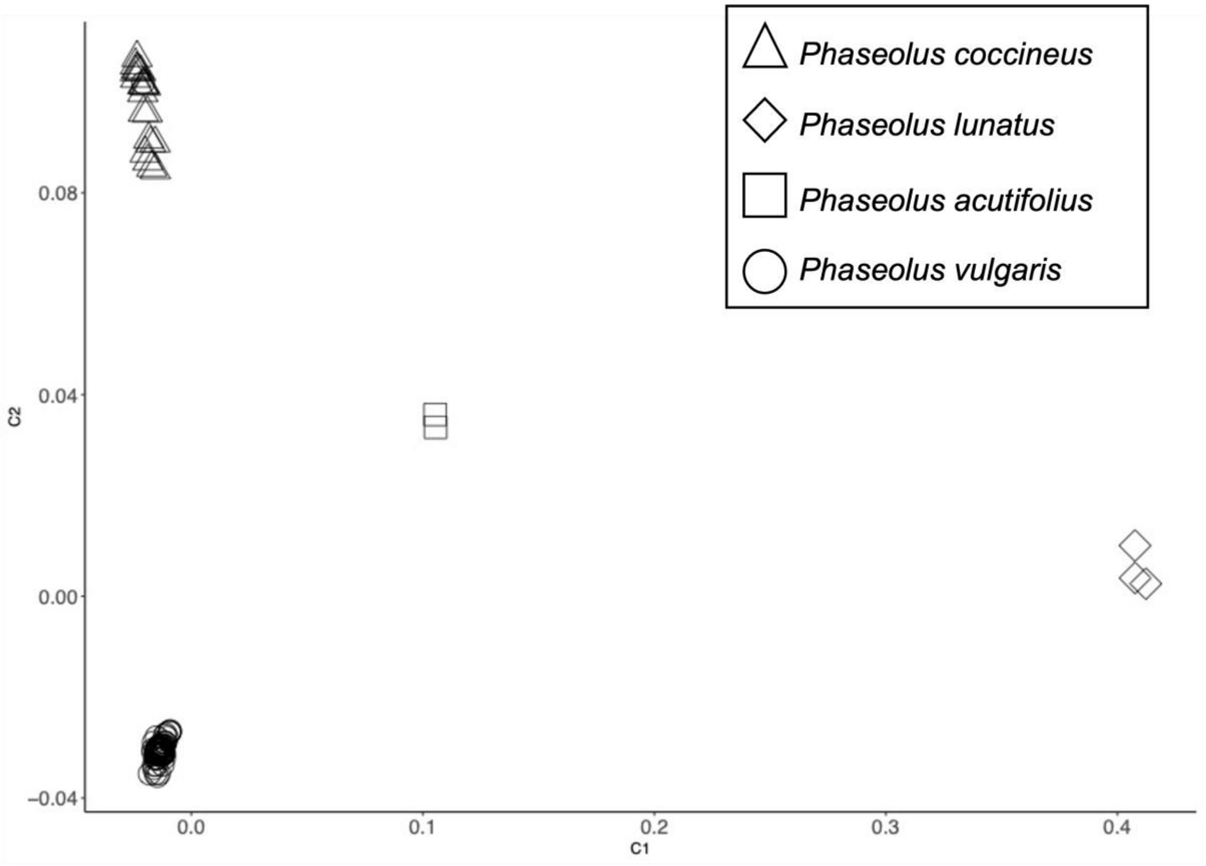

Supplementary_Figure_5: Results of the Multidimensional Scaling Analysis (MDS) of all accessions included in the analyses. Component C1 and C2 are reported in x and y axes.

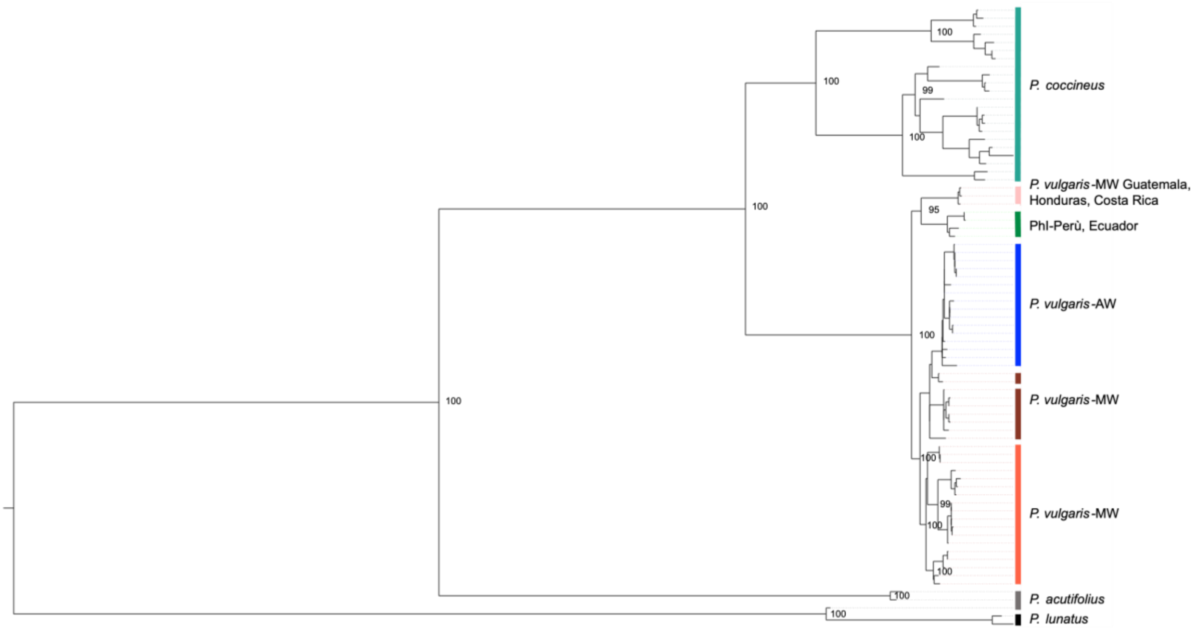

Supplementary_Figure_6: ML tree obtained from 3,231 SNPs collected from 97 samples of *Phaseolus* spp, bootstrap value=10,000.

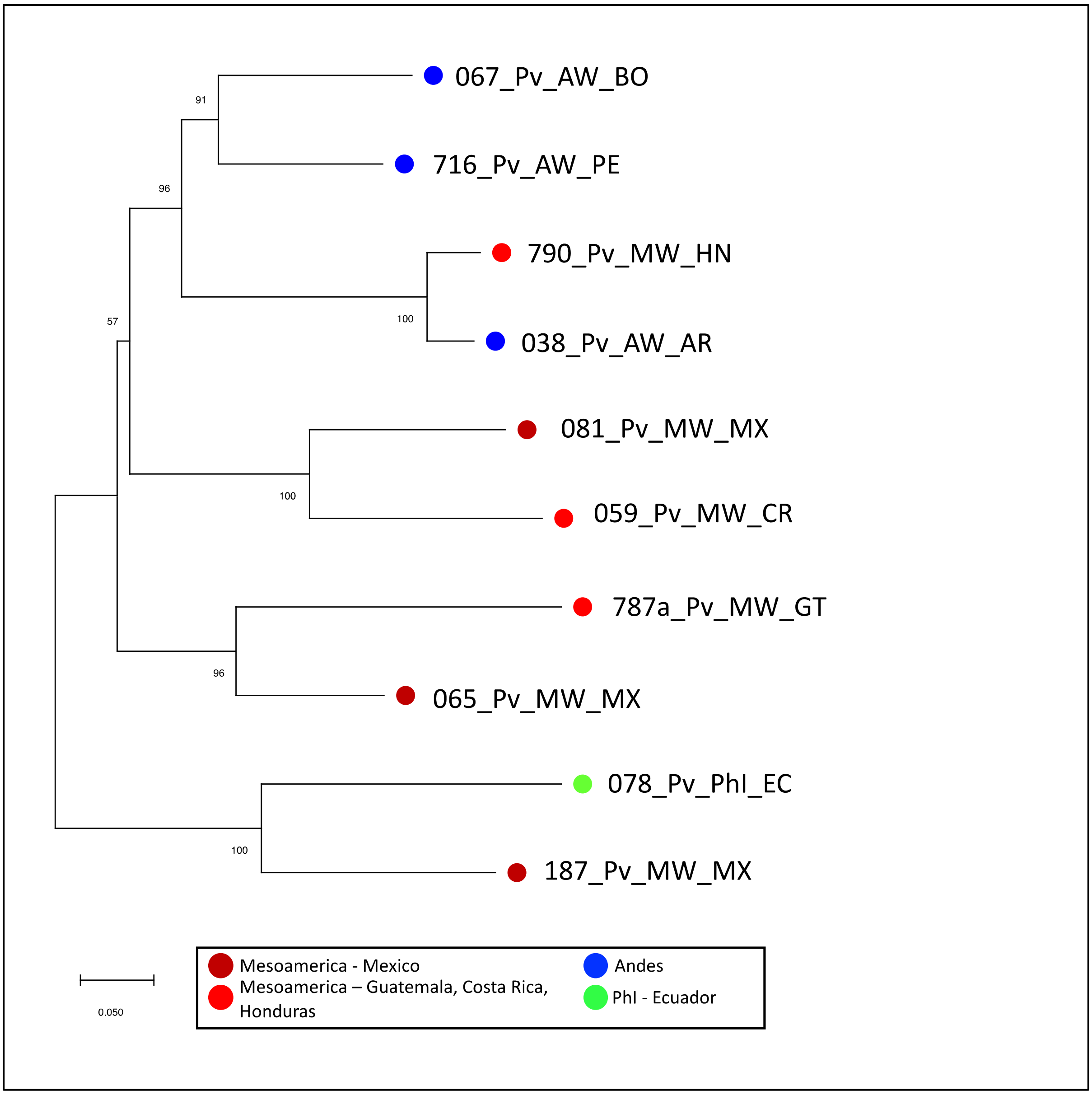

Supplementary_Figure_7: Neighbor Joining tree performed with nuclear SNPs selected every 250 kb with a bootstrap value of 10,000. In dark red: Mesoamerican samples from Mexico, light red: Mesoamerican samples from Guatemala, Costa Rica or Honduras, blue: Andean samples and in green: PhI sample from Ecuador.

Isolation and enrichment of phaseolin and SDS-PAGE analysis

To verify the presence of the phaseolin protein type I, 7 accessions were used, 5 of which are included in the present study (i.e., G11056, G19891, G19898, G23429, G23726) and the remaining two (i.e., G21245 and G21245A) were chosen from the CIAT database due to the documented presence of the phaseoline type I.

Seed flours were extracted with 20 volumes of protein extraction buffer (Sodium Borate buffer 180 mM pH9, NaCl 0.5 M) for 2h at room temperature and then were centrifuged at 20.000 g for 20 minutes at room temperature. The supernatant fraction was acidified with the addition of 1/80 volume of glacial acetic acid and kept at 4°C for 15 minutes. This caused immediate precipitation of phaseolin. The suspension was centrifuged at 20.000 g for 20 minutes at 4°C. The precipitate was directly dissolved in denaturation buffer (20 mM TRIS-HCl pH 8.6, 1% SDS, 0.6% □ME, 0.8% Glycerol), heat denatured at 100°C for 5 min and loaded on 15% SDS-PAGE as previously described by Bollini and Chrispeels (1978) Characterization and subcellular localization of vicilin and phytohemagglutinin, the two major reserve proteins of *Phaseolus vulgaris L.* [Planta 142:291-298].

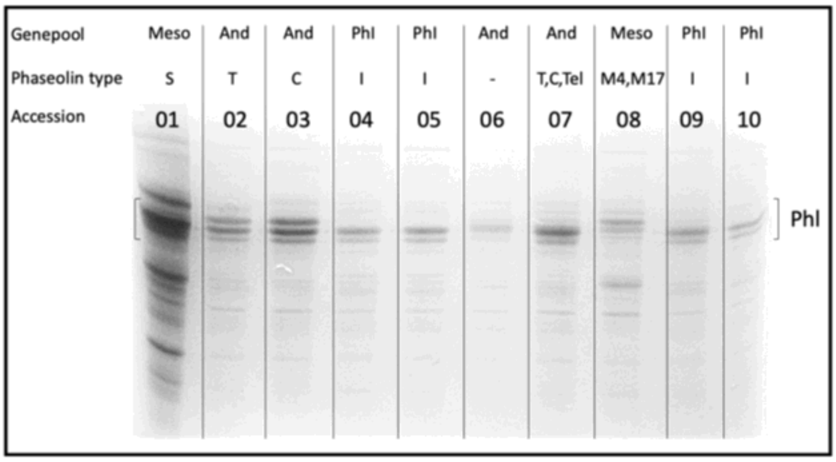

Supplementary_Figure_8: SDS-PAGE results. Accessions are listed as follow: 01, G11056; 02, G19891; 03, G19898; 04, G21245; 05, G21245; 06, G21245; 07, G21245A; 08, G23429; 09, G23726; 10, G23726.

Supplementary_Table_1: Panel of accessions selected to study the cpDNA.

Project code	Species	Accession Number	Country
P043_Pv_MW_GT	*P. vulgaris*	G19909	Guatemala
044_Pv_MW_MX	*P. vulgaris*	G20515	Mexico
P047_Pv_MW_CO	*P. vulgaris*	G21117	Colombia
069_Pv_MW_CO	*P. vulgaris*	G23462	Colombia
501_Pv_MW_MX	*P. vulgaris*	PI417671	Mexico
059_Pv_MW_CR	*P. vulgaris*	G23418	Costa Rica
746a_Pv_MW_MX	*P. vulgaris*	G12879	Mexico
787a_Pv_MW_GT	*P. vulgaris*	G23439	Guatemala
790_Pv_MW_HN	*P. vulgaris*	G50724	Honduras
887_Pv_MW_MX	*P. vulgaris*	NI1433	Mexico
911_Pv_MW_MX	*P. vulgaris*	86	Mexico
951_Pv_MW_MX	*P. vulgaris*	M31	Mexico
716_Pv_AW_PE	*P. vulgaris*	G23458	Peru
P832_Pv_MW_MX	*P. vulgaris*	G23470	Mexico
835_Pv_MW_MX	*P. vulgaris*	G23551	Mexico
id837fa1_Pv_MW_MX	*P. vulgaris*	G2771	Mexico
id837fa2_Pv_MW_MX	*P. vulgaris*	G11056	Mexico
id837fa3_Pv_MW_MX	*P. vulgaris*	G12957	Mexico
id837fa4_Pv_MW_MX	*P. vulgaris*	G13021	Mexico
id837fa7_Pv_MW_MX	*P. vulgaris*	G50899	Mexico
id837fa8_Pv_MW_MX	*P. vulgaris*	G12873	Mexico
007_Pv_MW_MX	*P. vulgaris*	G9989	Mexico
010_Pv_MW_MX	*P. vulgaris*	G11050	Mexico
011_Pv_MW_MX	*P. vulgaris*	G11051	Mexico
015_Pv_MW_MX	*P. vulgaris*	G12872	Mexico
016_Pv_MW_MX	*P. vulgaris*	G12877	Mexico
017_Pv_MW_MX	*P. vulgaris*	G12896	Mexico
019_Pv_MW_MX	*P. vulgaris*	G12922	Mexico
020_Pv_MW_MX	*P. vulgaris*	G12924	Mexico
021_Pv_MW_MX	*P. vulgaris*	G12927	Mexico
022_Pv_MW_MX	*P. vulgaris*	G12930	Mexico
028_Pv_MW_MX	*P. vulgaris*	G13505	Mexico
056_Pv_MW_CO	*P. vulgaris*	G22304	Colombia
057_Pv_MW_MX	*P. vulgaris*	G22837	Mexico
065_Pv_MW_MX	*P. vulgaris*	G23429	Mexico
076_Pv_MW_MX	*P. vulgaris*	G23652	Mexico
080_Pv_MW_MX	*P. vulgaris*	G24378	Mexico
081_Pv_MW_MX	*P. vulgaris*	G24571	Mexico

179_Pv_MW_MX	*P. vulgaris*	PI318696	Mexico
187_Pv_MW_MX	*P. vulgaris*	PI325677	Mexico
205_Pv_MW_MX	*P. vulgaris*	PI417775	Mexico
504_Pv_MW_MX	*P. vulgaris*	PI535430	Mexico
505_Pv_MW_MX	*P. vulgaris*	PI535409	Mexico
506_Pv_MW_MX	*P. vulgaris*	PI535450	Mexico
006_Pv_AW_AR	*P. vulgaris*	G7469	Argentina
031_Pv_AW_AR	*P. vulgaris*	G19888	Argentina
033_Pv_AW_AR	*P. vulgaris*	G19891	Argentina
038_Pv_AW_AR	*P. vulgaris*	G19897	Argentina
039_Pv_AW_AR	*P. vulgaris*	G19898	Argentina
052_Pv_AW_AR	*P. vulgaris*	G21199	Argentina
062_Pv_AW_PE	*P. vulgaris*	G23422	Peru
P064_Pv_AW_PE	*P. vulgaris*	G23426	Peru
066_Pv_AW_BO	*P. vulgaris*	G23444	Bolivia
067_Pv_AW_BO	*P. vulgaris*	G23445	Bolivia
068_Pv_AW_PE	*P. vulgaris*	G23455	Peru
232_Pv_AW_AR	*P. vulgaris*	W617499	Argentina
243_Pv_AW_BO	*P. vulgaris*	W618826	Bolivia
656_Pv_AW_AR	*P. vulgaris*	G19902	Argentina
665_Pv_AW_AR	*P. vulgaris*	NI1423	Argentina
668a_Pv_AW_BO	*P. vulgaris*	G23442	Bolivia
717_Pv_AW_PE	*P. vulgaris*	G23459	Peru
P718a_Pv_AW_PE	*P. vulgaris*	G23419	Peru
034_Pv_AW_AR	*P. vulgaris*	G19892	Argentina
040_Pv_AW_AR	*P. vulgaris*	G19901	Argentina
715_Pv_AW_PE	*P. vulgaris*	G23456A	Peru
013_Pv_AW_PE	*P. vulgaris*	G12856	Perù
id837fa6_Pv_PhI_EC	*P. vulgaris*	G23724	Ecuador
073_Pv_PhI_EC	*P. vulgaris*	G23582	Ecuador
075_Pv_PhI_PE	*P. vulgaris*	G23587	Perù
078_Pv_PhI_EC	*P. vulgaris*	G23726	Ecuador
Pc_1_W_MX	*P. coccineus*	PI430189	Messico
Pc_3_W_MX	*P. coccineus*	NI677	Mexico
Pc_4_W_MX	*P. coccineus*	NI819	Mexico
Pc_18_W_MX	*P. coccineus*	NI1120	Mexico
Pc_20_W_MX	*P. coccineus*	PI325598	Mexico
Pc_8_W_MX	*P. coccineus*	NI1092	Mexico
Pc_21_W_MX	*P. coccineus*	NI1117	Mexico
Pc_2_W_MX	*P. coccineus*	PI430183	Messico
Pc_5_W_MX	*P. coccineus*	NI1265	Mexico

Pc_6_W_MX	*P. coccineus*	PI417607	Mexico
Pc_7_W_MX	*P. coccineus*	PI417608	Mexico
Pc_9_W_MX	*P. coccineus*	PI417593	Messico
Pc_10_W_MX	*P. coccineus*	PI430178	Messico
Pc_11_W_MX	*P. coccineus*	PI346950	Messico
Pc_12_W_MX	*P. coccineus*	NI818	Mexico
Pc_13_W_MX	*P. coccineus*	NI1213	Mexico
Pc_14_W_MX	*P. coccineus*	NI726	Mexico
Pc_15_W_MX	*P. coccineus*	NI813	Mexico
Pc_16_W_MX	*P. coccineus*	NI1122	Mexico
Pc_17_W_MX	*P. coccineus*	NI1028	Mexico
Pc_19_W_MX	*P. coccineus*	NI1125	Mexico
Pc_22_W_MX	*P. coccineus*	NI1325	Mexico
Pa_D_SV	*P. acutifolius*	PI200902	El Salvador
Pa_W_MX	*P. acutifolius var. acutifolius*	PI319445	Mexico
Pl_D_MX	*P. lunatus*	PI313212	Mexico
Pl_W_GT	*P. lunatus*	NI1689	Guatemala
Pl_W_PE	*P. lunatus*	NI1771	Peru

Supplementary_Table_2: Panel of accessions selected to study the nuclear DNA.

Project code	Species	Gene-pool	Country
187_Pv_MW_MX	*P. vulgaris*	Mesoamerican Wild	Mexico (Morelos)
065_Pv_MW_MX	*P. vulgaris*	Mesoamerican Wild	Mexico (Puebla)
081_Pv_MW_MX	*P. vulgaris*	Mesoamerican Wild	Mexico (Oaxaca)
787A_Pv_MW_GT	*P. vulgaris*	Mesoamerican Wild	Guatemala (Santa Rosa)
790_Pv_MW_HN	*P. vulgaris*	Mesoamerican Wild	Honduras (El Paraiso)
59_Pv_MW_CR	*P. vulgaris*	Mesoamerican Wild	Costa Rica (San Jose)
038_Pv_AW_AR	*P. vulgaris*	Andean Wild	Argentina (Tucuman)
067_Pv_AW_BO	*P. vulgaris*	Andean Wild	Bolivia (Tarija)
716_Pv_AW_PE	*P. vulgaris*	Andean Wild	Peru (Cuzco)
078_Pv_PhI_EC	*P. vulgaris*	North Peru-Ecuador (PhI)	Ecuador (Chimborazo)

Supplementary_Table_3: Centromeric regions and corresponding number of analyzed SNPs.

Chr	Centromeres Dimensions (Mb)	N. SNPs
1	7.7	170,779
2	4.6	113,647
3	2.1	46,248
4	6.5	163,594
5	7.5	173,932
6	0.1	2,803
7	13.6	334,210
8	13.9	294,893
9	4.3	109,616
10	0.7	16,317
11	1.0	24,544

References

Bollini R., and M. J. Chrispeels. "Characterization and subcellular localization of vicilin and phytohemagglutinin, the two major reserve proteins of Phaseolus vulgaris L." Planta 142.3 (1978): 291-298.

Darling A. C., B. Mau, F. R. Blattner, N. T. Perna. Mauve: multiple alignment of conserved genomic sequence with rearrangements. Genome Res. 2004 Jul;14(7):1394-403. doi: 10.1101/gr.2289704. PMID: 15231754; PMCID: PMC442156.

Frazer K. A., L. Pachter, A. Poliakov, E. M. Rubin, I. Dubchak. VISTA: computational tools for comparative genomics. Nucleic Acids Res. 2004 Jul 1;32(Web Server issue):W273-9

Katoh K., J. Rozewicki, K. D. Yamada. MAFFT online service: multiple sequence alignment, interactive sequence choice and visualization, Briefings in Bioinformatics, Volume 20, Issue 4, July 2019, Pages 1160–1166, https://doi.org/10.1093/bib/bbx108

Rozas J., A. Ferrer-Mata, J. C. Sánchez-DelBarrio, S. Guirao-Rico, P. Librado, S. E. Ramos-Onsins, A. Sánchez-Gracia (2017). DnaSP 6: DNA Sequence Polymorphism Analysis of Large Datasets. Mol. Biol. Evol. 34: 3299-3302. DOI: 10.1093/molbev/msx248
